# Interobserver reliability of the pivot shift test: A modified classification improves agreement

**DOI:** 10.1002/jeo2.70354

**Published:** 2025-08-05

**Authors:** Juan P. Martinez‐Cano, Sebastian Mejia‐Barreto, Jacobo Triviño‐Arias, Maria C. Gomez‐Ayala, Alejandro Mejia, Juan F. Londoño, Ruben Guzman

**Affiliations:** ^1^ Departamento de Ortopedia Fundación Valle del Lili Cali Colombia; ^2^ Facultad de Ciencias de la Salud Universidad Icesi Cali Colombia; ^3^ Hospital Universitario de la Samaritana Bogotá Colombia; ^4^ Fundación Valle del Lili, Centro de Investigaciones Clínicas Cali Colombia; ^5^ Universidad Pontificia Bolivariana Medellín Colombia; ^6^ Clínica El Rosario Medellín Colombia

**Keywords:** anterior cruciate ligament, diagnostic test, joint instability, knee, knee injury, physical examination

## Abstract

**Purpose:**

The pivot shift test evaluates the anterolateral rotational instability of the knee in patients with anterior cruciate ligament (ACL) injuries. The aim of this study was to evaluate the interobserver reliability of the classic pivot shift test and a modified classification.

**Methods:**

An interobserver reliability study involving 4 observers and 17 patients with high suspicion of ACL injury. Observers were blind to diagnostic images and independently evaluated each patient. Kappa–Fleiss was used to assess the pivot shift test agreement between observers. Interobserver reliability was calculated for the classic classification (grades I, II and III), as well as for a modified classification in low‐grade (I) and high‐grade pivot shift (II and III). Kappa agreement was categorised as poor (<0.00), slight (0.00–0.20), fair (0.21–0.40), moderate (0.41–0.60), substantial (0.61–0.80) and perfect (0.81–1.00).

**Results:**

Patients had mean age of 29 years (range: 24–32) with similar sex distribution (male: 53%). The global interobserver reliability assessment favoured the modified (0.73) over the classic classification (0.39). Agreement for the classic categories (negative: 0.87, grade I: 0.61, grade II: 0.06 and grade III: 0.34), was surpassed by the modified approach (negative: 0.87, low‐grade: 0.61 and high grade: 0.73).

**Conclusion:**

Our study demonstrated that the modified pivot shift test grading improves interobserver reliability compared to the classic classification. By combining the grade II and III categories into a single high‐grade category, we transformed poor and fair agreement into substantial agreement. The modified pivot shift classification can be utilised in both clinical practice and research, particularly due to its clinical implications for decision‐making in patients with grade II and III pivot shifts, which may be similar.

**Level of Evidence:**

Level I.

**Abbreviations**
ACL: anterior cruciate ligament
ACLR: anterior cruciate ligament reconstruction
ALL: anterolateral ligament
IRB: institutional review board
LET: lateral extraarticular tenodesis
PCL: posterior cruciate ligament
SD: standard deviation
VSM: vector polynomial support machine

## INTRODUCTION

Anterior cruciate ligament (ACL) tears are among the most common knee injuries [4]. ACL reconstruction (ACLR) is one of the most frequently performed orthopaedic procedures worldwide, with estimated annual costs in the United States reaching approximately $1 billion in 2006 [14]. Clinical diagnosis of ACL injuries can be achieved with over 90% accuracy through a combination of patient history and physical examination [5]. The anterior drawer, Lachman and pivot shift tests have reported sensitivities of 79.6%, 98.6% and 89.8%, respectively, for detecting ACL tears [17]. While the anterior drawer and Lachman tests assess anterior tibial translation, the pivot shift test specifically evaluates the anterolateral rotational instability characteristic of ACL‐deficient knees [16].

ACLR aims to recover functional stability of the knee and help patients to return to their preinjury functionality [16]. Some patients may persist with anterolateral rotatory laxity and residual pivot shift after surgery, with worse postoperative outcomes and subjective instability [1, 3, 12, 13]. In an attempt to better control rotational stability and reduce graft re‐rupture, anterolateral ligament (ALL) reconstruction or lateral extra‐articular tenodesis (LET) has been proposed and implemented in selected patients [6, 7, 27]. The severity of pivot shift has been suggested by some authors as a key point in the decision making for adding or not an extra‐articular anterolateral procedure [28, 29].

The pivot shift test was described in 1972 by Galway et al. as a clinical sign of symptomatic anterior cruciate insufficiency [4]. Three grades of pivot shift have been defined, but there are differences in the way the manoeuvre is performed and graded by different examiners [12, 25]. The subjectivity of grading has been questioned and devices that quantify objectively the pivot shift have been designed [21]. Though, most are not available for clinical use yet and the traditional pivot shift test in the office is the way in which most orthopaedic surgeons continue assessing ACL anterolateral rotational instability

There are no studies that have assessed the interobserver reliability for the different grades of the pivot shift test, only for a positive or negative test [26]. This study aims to evaluate the interobserver reliability of the classic pivot shift test and a modified classification.

## METHODS

### Type of study and participants

This was a cross‐sectional, reliability study, performed in September 2020. Eligibility criteria included patients between 10 and 70 years, with high suspicion of ACL tear or re‐tear (clinically plus magnetic resonance imaging); diagnosis was further confirmed by arthroscopic assessment. Multiligament knee injuries were excluded. Patients were sequentially recruited by convenience. The study was approved by the institutional review board (IRB) from Clinica El Rosario, where the research was conducted.

### Pivot shift grading and manoeuvre

ACL rotational laxity of each knee was evaluated by four examiners (orthopaedic sports medicine surgeons, with similar experience, within 10 years in practice) with the pivot shift test. All of them received the same standardised training in the pivot shift manoeuvre and in the classic clinical classification, prior to the study initiation. The observers were blind to medical chart, magnetic resonance imaging and to each other's results during the study. Each of the four raters performed the test once, consecutively on each patient, within the same day, in the office.

The definition that was used to classify the pivot shit test was: grade 0: no displacement with the manoeuvre; grade I: subtle glide of the knee; II: an intermediate movement between subtle glide or an explosive displacement; III: gross or explosive movement of the knee [4, 9, 25]. The pivot shift manoeuvre was instructed to be performed in three steps as described by Musahl et al.: Step 1: the examiner controls the patient's leg with its ipsilateral hand at the heel level, lifts the patient's leg and internally rotates the tibia with the ipsilateral hand. Step 2: the examiner's contralateral hand is placed on the lateral side of the knee. A gentle valgus stress is applied; the knee is naturally flexed with the combined stress of internal rotation and valgus stress. Step 3: knee flexion is advanced with both hands, internal rotation and valgus are maintained until 20 degrees of knee flexion and at the point of shifting, the rotational stress of the ipsilateral hand is released and the proximal tibia is guided into external rotation by the contralateral hand, which accentuates the reduction movement [25].

A modified classification was used for this study, with only two categories: low‐grade (grade I) and high‐grade (grouping grades II and III). Grades II and III were grouped as *high‐*grade based on the premise that both represent a clinically significant anterolateral rotational instability that has decision‐making implications as they might need to be surgically assessed. As a secondary objective, it was planned to compare interobserver reliability between both ways of grading.

### Statistical analysis

A descriptive statistical analysis was carried out, quantitative variables are expressed as mean and standard deviation (SD), or median and interquartile range, and categorical variables are presented in relative and absolute frequencies. Kappa–Fleiss was used to assess the pivot shift test agreement between the four orthopaedic surgeons. Kappa–Fleiss is an index to evaluate the reliability of the interrater agreement. Kappa agreement values were categorised as poor (<0.00), slight (0.00–0.20), fair (0.21–0.40), moderate (0.41–0.60), substantial (0.61–0.80) and perfect (0.81–1.00) [19].

The sample size calculation was based on the Kappa–Fleiss agreement, with a set alpha error of 5% and power of 80%. Assuming a minimally acceptable Kappa–Fleiss of 0.5 and an expected agreement of 0.8, the calculated required sample size was 15 participants. Anticipating a potential attrition rate of 10%, the final target sample size was increased to 17 participants. All analyses were performed using Stata version 14 (StataCorp LP).

## RESULTS

The study included 17 participants (17 knees). The mean age was 29 years with similar sex distribution (Table 1). Regarding the mechanism of trauma, eight(47.1%) corresponded to sports injuries, eight (47.1%) to traffic accidents and one (5.9%) to falls. Concerning the patient diagnostic, eight (47.1%) were ACL rupture, eight (47.1%) were ACL re‐rupture and one (5.9%) was posterior cruciate ligament (PCL) rupture. Moreover, four of the patients had associated lesions. About the physical examination, 15 had a positive Lachman's sign, and four complained of pain and limitation.

**Table 1:** Baseline characteristics of participants.

Variable	*n* = 17
Age^a^ (years)	29 (24–32)
Sex	
Male	9 (52.9%)
Female	8 (47.1%)
Trauma mechanism	
Sports	8 (47.1%)
Car accident	8 (47.1%)
Falls	1 (5.9%)
Laterality	
Right	13 (76.5%)
Left	4 (23.5%)
Time since injury (months), mean (SD)	3.8 (3.1)
Diagnosis	
ACL rupture	8 (47.1%)
ACL re‐rupture	8 (47.1%)
PCL rupture	1 (5.9%)
Associated injury	
No	13 (76.5%)
Yes	4 (23.5%)
Lachman	
Negative	2 (11.8%)
Positive	15 (88.2%)
Pain/limitation on physical examination	
No	13 (76.5%)
Yes	4 (23.5%)

Abbreviations: ACL, anterior cruciate ligament; PCL, posterior cruciate ligament; SD, standard deviation.

a Median (interquartile range).

### Interobserver reliability

The pivot shift classification interobserver reliability graded from 0 to 3 (classic pivot shift) was evaluated (Table 2). The results showed a fair general Kappa–Fleiss of 0.391. When discriminated by categories, a perfect, substantial and moderate agreement was respectively found for categories 0, 1 and 3 (0.872, 0.609 and 0.339). The worst agreement was seen in category 2 (0.056), which was poor. The modified classification, demonstrated a substantial agreement with 0.734, with Kappa–Fleiss; the different categories showed perfect agreement for negative pivot shift (0.872) and substantial agreement for low grade (0.609) and high‐grade (0.732).

**Table 2:** Interobserver reliability for pivot shift test grading.

Classic Pivot shift classification			Modified classification		
	Kappa–Fleiss	95% confidence interval (CI)		Kappa–Fleiss	IC 95%
General	0.391	0.27–0.51	General	0.734	0.59–0.88
Grade 0	0.872	0.68–1	Negative	0.872	0.69–1
Grade I	0.609	0.42–0.8	Low‐grade	0.609	0.42–0.80
Grade II	0.056	0.14–0.25	High‐grade	0.732	0.54–0.93
Grade III	0.339	0.14–0.53			

## DISCUSSION

The major findings of our study showed that a modified pivot shift grading system improves the interobserver reliability. There was an improvement in agreement for both the general agreement of the classification (from 0.39 to 0.73), and for the individual categories. This was a consequence of unifying grades II and III from the classic classification into a high‐grade pivot shift category.

The lowest reliability was observed in grade II (0.06) and grade III (0.34), which improved to substantial agreement when categorised together as high‐grade pivot shift (0.73). This suggests that clinicians face challenges in distinguishing between grade II and grade III categories, particularly with grade II, which showed poor agreement. The difficulties associated with grade II may stem from its position between a subtle and an explosive movement, lacking a distinct characteristic that would facilitate its easy differentiation. In contrast, it is straightforward to identify when a patient exhibits no pivot shift (perfect agreement) or a grade I pivot shift (substantial agreement). Furthermore, differentiating between grade I (low‐grade) and grades II–III (high‐grade) is both simple and reliable.

It is a key issue to evaluate the anterolateral rotational instability before and after performing an ACL reconstruction surgery. Persistent pivot shift (anterolateral rotational instability) remains as a problem in some patients after surgery, associating with inferior outcomes [1, 13, 20]. Therefore, it is desirable that this clinical test would have substantial agreement between different observers, producing more reliable data in clinical practice and research.

A postoperative positive pivot shift has been associated with more symptoms of instability and a higher risk of knee osteoarthritis after ACLR [18, 23]. Gupta et al., found in a cohort of 362 patients, that a preoperative high‐grade pivot shift test increases the likelihood of worse functional outcomes and has lower rate of return to sport [8]. Kamada et al., studied 164 patients operated with double bundle ACLR, and reported persistent postoperative pivot‐shift in 8.5% of cases; the risk factors associated were younger age (<20 years), high‐grade preoperative pivot shift and knee hyperextension [15].

In order to reduce residual pivot shift (anterolateral rotational instability), additional procedures for augmentation of the anterolateral complex of the knee, such as LET or ALL reconstruction, have been proposed [30]. In fact, having a grade II or III pivot shift (high‐grade pivot shift in the modified classification), is one of the indications to consider this augmentation [29]. Patients with this high‐grade of pivot shift benefit from anterolateral augmentation; Getgood et al., studied 618 patients with ACL injury in the STABILITY study (randomised controlled trial), demonstrating lower rate of clinical failure (graft rupture and residual positive pivot‐shift) at 2 years after surgery, in the group treated with ACLR plus LET, compared with the ACLR group [7]. This shows that the pivot shift is not only useful in the decision‐making process for performing an augmentation in ACLR surgery, but also as an outcome variable to be measured during follow‐up.

Multiple devices have been designed aiming to quantify objectively knee laxity, including anterolateral rotation [2, 10, 11, 24]. A recent systematic review, described that instrument‐based assessment of anterolateral rotatory knee laxity have reported an interobserver reliability between 0.63 and 0.99 [22]. Evaluating anterolateral instability in ACL‐deficient knees with these devices could help decision‐making when considering anterolateral augmentation. However, the use of these devices in the office is limited because they are not easily available. That's why it is very important to understand better the pivot shift interobserver reliability and find ways of improving it, as this study shows. We propose a modified classification for grading the pivot shift test with better interobserver reliability compared to the classic classification.

Our research has some limitations. The tests were conducted by knee surgeons with experience in evaluating knees, therefore, the results may not extrapolate to less experienced healthcare professionals. However, the pivot shift test is predominantly utilised worldwide by knee surgeons with characteristics like those of the observers included in this study. Additionally, the sample size may be considered small, as it comprised only 17 knees. Nevertheless, there were four observers and 68 measurements, which were in accordance with the sample size calculation and deemed sufficient for statistical analysis in this reliability study.

## CONCLUSION

Our study demonstrated that the modified pivot shift test grading improves interobserver reliability compared to the classic classification. By combining the grade II and III categories into a single high‐grade category, we transformed poor and fair agreement into substantial agreement. The modified pivot shift classification can be utilised in both clinical practice and research, particularly due to its clinical implications for decision‐making in patients with grade II and III pivot shifts, which may be similar.

## AUTHOR CONTRIBUTIONS

**Juan P. Martinez‐Cano**: Conceptualisation and research question development; literature review; writing; drafting; methodological review. **Sebastian Mejia‐Barreto**: Literature review; writing; drafting and manuscript review. **Jacobo Triviño‐Arias**: Literature review; writing; drafting and manuscript review. **Maria C. Gomez‐Ayala**: Literature review; writing; drafting; manuscript review and methodological review. **Alejandro Mejia**: Literature review; writing; drafting and manuscript review. **Juan F. Londoño**: Literature review; writing; drafting and manuscript review. **Ruben Guzman**: Literature review; writing; drafting and manuscript review.

## CONFLICT OF INTEREST STATEMENT

The authors declare no conflicts of interest.

## ETHICS STATEMENT

The study was approved by the institutional review board (IRB) of Clinica El Rosario.

## Data Availability

The data that support the findings of this study are available on request from the corresponding author. The data are not publicly available due to privacy or ethical restrictions.
